# Evaluation of the impact of the educational revolution in Peru and the gender wage gap, 2017-2021

**DOI:** 10.12688/f1000research.153475.1

**Published:** 2024-08-05

**Authors:** Percy Junior Castro Mejía, Rogger Orlando Morán-Santamaría, Yefferson Llonto Caicedo, Francisco Eduardo Cúneo Fernández, Nikolays Pedro Lizana Guevara, Hilda Paola Arias Gonzales, Lindon Vela Meléndez

**Affiliations:** 1 La Libertad, Cesar Vallejo University, Trujillo, La Libertad, Peru; 2 Lambayeque, Universidad Nacional Pedro Ruiz Gallo, Lambayeque, Lambayeque, Peru

**Keywords:** wage gap, income, education, educational policy, Tobit model

## Abstract

**Background:**

Women’s educational attainment and their generation of value through education has increased the prospects for achieving economic equality between men and women. However, women continue to earn lower wages than men, reflecting growing inequality in several countries. Therefore, the objective of the study is to estimate the impact of education on the gender wage gap in Peru over the period 2017-2021.

**Methods:**

Quantitative, explanatory study aimed at identifying the impact of education on the gender wage gap in Peru during the period 2017-2021. The research design is non-experimental and uses a time series that analyses the influence of the latent variable of education on the gender wage gap. This is a continuous variable to estimate the Tobit model.

**Results:**

The results show that the gender gap in Peru exhibited a decreasing trend between men and women during the period 2017-2020, with an average reduction of 10% until 2020 due to the health crisis. The highest average salary was achieved by men in 2019, reaching S/2289.97 soles, while women reached an average salary of S/1368.85 soles. In the post-pandemic scenario for 2021, the gender gap increased by 3%, with men earning an average salary of S/1999.63 soles and women earning an average salary of S/1281.16 soles. The analysis from 2017-2021 shows that years of education had a positive impact on the gender wage gap in Peru based on the Tobit model estimation.

**Conclusions:**

During the analysis period of 2017-2021, years of education had a positive impact on the gender wage gap in Peru, with the greatest impact occurring during the health crisis. The probability of women’s incomes improving with an increase in years of education was 2.35%, while for men, the highest impact was in 2018, with a probability of income improvement of 2.16% in terms of marginal effect.

## 1. Introduction

The health crisis highlighted the need for a new social contract that addresses the poor distribution of surplus involving wages, salaries, or dividends from the perspective of Social Economy. Following the health crisis, inequalities were exacerbated due to existing poor redistributive mechanisms ( Costas, 2020; Gonzáles, 2021; Singh et al., 2022; Nguyen, 2022; Abdel et al., 2023). It can be observed that the pandemic context fosters situations where income disparities between men and women in developing and developed countries persist despite women’s growing educational attainment ( Kireyeva & Satybaldin, 2019; Brzezinski, 2021; Da Costa & Shinkoda, 2021; Afrin & Shammi, 2023).

The educational achievement of women and their value generation through education has led to the prospect of achieving economic equality between men and women. However, women continue to receive lower wages compared to men, reflecting growing inequality in countries such as the United States and Latin American countries. Education is the main factor for women in achieving significant economic success in wage discrimination ( Kurek & Górowski, 2020; Litman et al., 2020; England et al., 2020; Reissová & Šimsová, 2019).

There is extensive literature on the persistent gender wage gap in various countries and economic sectors, expressed in working hours, experience, years of education, salary, and labor market. However, there are few studies on the expected return of education on the gender wage gap, which has a significant impact on wage disparity ( Fox et al., 2019; Ñiquen, 2019; Smith et al., 2021).

According to Suharyono and Digdowiseiso (2021), in theoretical evidence, salary is the main motivation for labor effort. Empirical evidence has shown a positive effect of education on wage equality in a complex globalization context, where the indecisiveness of public policy managers highlights a concern for the obstacle in hindering women’s continuous development and other associated factors such as stereotypes and ideology restricting women’s access to education ( Quadlin et al., 2023; Leibing et al., 2023).

For the International Labour Organization (ILO) in Latin America and the Caribbean, women have had greater participation in the labor aspect in recent decades. However, there is still a 25% economic dependency gap of women on men ( Dancausa Millán et al., 2021; Organización Internacional de Trabajo, 2019).

In Peru, simply being a woman evidences the conditioning of receiving different wages, further accentuating the differentiation after the restrictions imposed by the COVID-19 health crisis. Despite the monthly wage gap narrowing from over 20% to 14% since 2016, and the gender gap in hourly wages decreasing from 13.9% to 9.2% in 2010 and 2016, this wage gap is associated with gender disparities related to the labor market’s own dynamics, where women have their own characterization related to variables such as sex, age, marital status, access to health services, among others. Education, along with women’s socioeconomic conditions, influences the probability of having better human capital ( Carlosviza et al., 2021; Defensoría del Pueblo, 2019; Rios, 2019; Valdez & Sobrevilla, 2021).

The role of women in economic development is often doubtful, as they are considered inferior, unworthy, and incapable of working. Gender discrimination reduces the economy’s growth capacity and the ability to improve the standard of living ( Schober & Winter-Ebmer, 2011; Sugiharti & Kurnia, 2018; Didier, 2021; Alwago, 2023; Bataka, 2024).

Salary is considered a broad topic in economic literature, as it involves both capabilities and skills that result in productivity indicators. It is necessary to point out that the return on education concerning wages is the compensation for education and experience, which starts from different points of the wage distribution. Therefore, the gender wage gap varies across different economic sectors ( Castagnetti & Giorgetti, 2019; Tansel et al., 2020; Mandel & Rotman, 2021).

It is essential to indicate that education has generated higher returns on income, validating the theory of investment in education as an investment in human capital. In the case of women, this has led to wage gaps reducing as they gain greater access to education ( Barra, 2018; Carlosviza et al., 2021).

Women and men have almost identical human capital; however, the gender wage gap and the trajectory it has created over time in various traditional labor markets have reflected the cumulative effects and gender roles that have emerged throughout life. The drivers of the mentioned gap are productivity factors such as education, skills, and work experience. These individual-level factors most representatively explain the gender wage gap ( Chowdhury et al., 2018; Designed & Performed, 2010; Díaz, 2021; Litman et al., 2020; Sterling et al., 2020).

In this context, the present study aims to estimate the impact of education on the gender wage gap in Peru during the period 2017-2021, for which the Tobit estimation, known as the Censored Regression Model, is performed.

In the review of scientific literature, the research by Picatoste et al. (2023) evidenced that the main concerns related to the gender gap are access, use, and outcomes, as well as the wage disparity existing in the European Union. Additionally, using mean comparison, it was shown that the variables significantly influencing the wage gap are related to educational level and social aspects. Meanwhile et al. (2023) in their findings, show that the gender wage gap significantly impacts women’s empowerment due to limitations in women’s economic independence and autonomy.

On the other hand, Penner et al. (2022) in their various studies, indicate that the gender wage gap highlights that various countries can be identified where the wage gap is a crucial aspect of gender differences due to the different jobs that substantially represent wage differences. Using the ordinary least squares methodology, four models related to wage disparity processes between men and women are compared, leading to a gender wage inequality that presents various challenges for the relevance of the 15 countries where the models’ results are compared. However, education is relevant not only as a factor explaining wage inequality between men and women but also between whites and blacks, implying the racist effect on racial inequality disparity, which increases the gap in the labor market with educational interventions that close racial disparities ( Zhou & Pan, 2023).

Thus, growing economic inequality has highlighted a variety of literature where wage dispersion involves significant inequality using the VECM model. Considering the structural model reveals that it affects inequality with various impacts on the labor market and becomes relevant in wages with a biased technological shock and structural inequality that negatively impacts working hours and reduces productivity by facing negative inequality in the labor market characterized by high levels of inequality ( Hutter & Weber, 2023).

Additionally, in American companies, data shows that female executives represent 6% of the sample, and 26% of women earn less than men. This is given the relevant characteristics where the existence of gender wage gaps within companies is evident, considering the marked income inequality estimated with the entry and exit rate equation methodology, which examines wage gaps ( Keller et al., 2023).

Finally, in Europe, it is still evident that women perceive higher levels of income inequality, revealing the persistence of gender wage disparities. In 15 of the 28 countries analyzed, women report the wage gap between incomes and expenditures, highlighting the driving force needed to reduce the gender wage gap and showing a persistence of these disparities ( Adriaans & Targa, 2023).

## 2. Methods

### 2.1 Methodological design

A quantitative approach is considered, with an explanatory type of research, confirmed by Maxwell and Reybold (2015) aimed at identifying the impact of education on the gender wage gap in Peru during the period 2017-2021. The research design is non-experimental ( Aarsman et al., 2024) and utilizes a time series analysis based on the influence of the latent variable of education on the gender wage gap, which is a continuous variable for estimating the Tobit model.

$${\mathrm{Yi}}^{*}=X_{i}\beta +\mathrm{\mu i}$$

$$\mathrm{yi}=\{0,\mathrm{si}\;{\mathrm{yi}}^{*}\leq 0{\mathrm{yi}}^{*},\mathrm{si}\;{\mathrm{yi}}^{*}\geq 0$$

Where:

yi: wage income of men and women in Peru during the period 2017-2021

Xi: explanatory variables such as age, number of children under 6 years old, years of education, and children aged between 6 and 18 years.

The Tobit model considered takes a fixed value for yi ≤ 0y_i \leq 0yi ≤ 0, given that there are men and women who decide not to work in the labor market, and that there are men and women with a salary equal to 0. The decision not to work corresponds to the inherent structure of the Peruvian labor market.

### 2.2 Procedure

The data were obtained from the data files of the National Household Survey from 2017 to 2021, specifically from Module 100: Housing and Household Characteristics, Module 200: Household Members’ Characteristics, Module 300: Education, and Module 500: Employment and Income. This data was published on the website of the Institute of the National Institute of Statistics and Informatics (INEI), where the information is freely accessible in accordance with the policies of the Peruvian state as a public entity.

Subsequently, the database was analyzed using the Stata 16 software. Explanatory variables such as age, number of children under 6 years old, years of education, and children aged between 6 and 18 years were used for estimating the Tobit model and identifying the impact of education on the gender wage gap in Peru during the period 2017-2021.

### 2.3 Sample

The sample includes the number of observations comprising dependent and independent men and women who earn income and are part of the economically active employed population. The inclusion and exclusion criteria are detailed below in Table 1.

**Figure 1.**
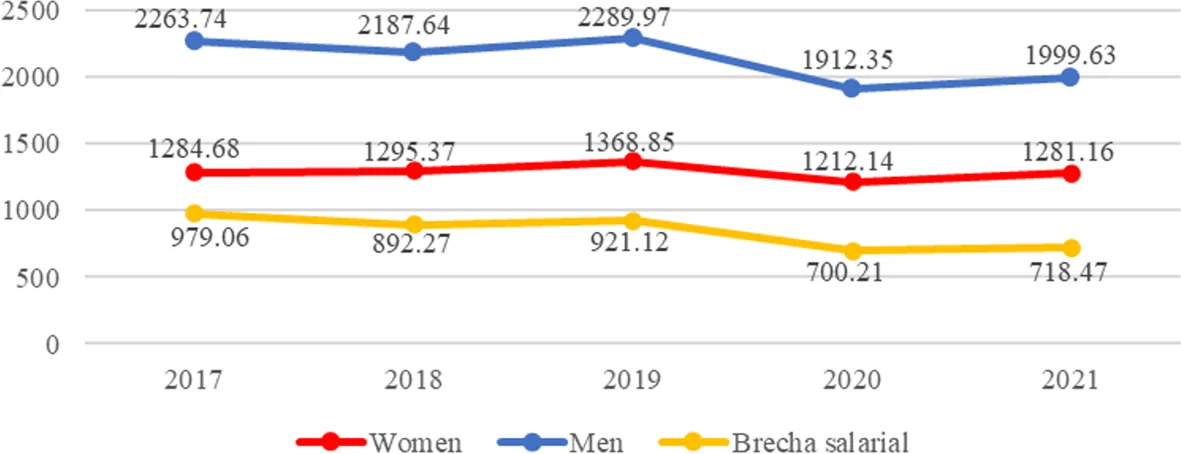
Dynamics of the Gender Wage Gap in the Period 2017-2021. Note: Values from the Encuesta Nacional de Hogares database, 2017-2021, obtained from INEI (2024).

**Table 1.** Sample for the period 2017-2021.

Year	Gender	
	Men	Women
2017	15313	4429
2018	15953	4791
2019	14350	4613
2020	13127	4352
2021	12678	5251

Note: Using Stata portable 16, 2022, presents data from ENAHO 2017-2021, obtained from INEI (2024).

**Table 2.** Data sources for the variables used in the methodology of this study.

Variables	Type	Description	Unit	Data sources
W_total	Dependent	Monthly income of men and women	Nuevos soles	Household Survey conducted by the National Institute of Statistics and Informatics (INEI)
Age	Explanatory of interest	Age	Years	
Minor_6	Explanatory of interest	Number of children under 6 years old	Unit	
Between_6_18	Explanatory of interest	Number of children aged between 6 and 18	Unit	
Educ	Explanatory of interest	Years of education	Years	

Note: Own structured description of the data for each of the variables of the study according to the methodology, obtained from INEI (2024).

**Inclusion criteria:** Men and women aged 14 and older who are part of the economically active employed population, both dependent and independent, and who earn income during the period 2017-2021.

**Exclusion criteria:** Men and women aged 14 and older who are part of the economically inactive population and the unemployed during the period 2017-2021.

### 2.4 Data analysis

The econometric estimation model Tobit was used to identify the impact of education on the gender wage gap in Peru during the period 2017-2021.

In this way, Stata 16 was used to estimate the monthly income gap between women and men in Peru for the year 2021.

The Tobit econometric model was used to identify the impact of education on the gender wage gap in Peru over the period 2017-2021. This model, implemented in Stata 16, allows us to address the censoring present in the monthly income data, correcting for possible biases and providing more precise and robust estimates. In this study, the dependent variable, monthly income, was bounded at zero for unemployed individuals. The use of Tobit allowed us to properly analyse this situation, ensuring the validity and reliability of the results obtained to better understand the relationship between education and the gender wage gap in Peru.

There is an academic licence for the use of Stata 16 software, registered in the name of Lindon Vela Meléndez. The details of the licence are as follows Serial number: 501809389697. The software can be downloaded from the following link: https://download.stata.com.

### 2.5 Ethical considerations

The data for this study come from the National Household Survey (ENAHO) of the National Institute of Statistics and Informatics (INEI), a public and anonymised dataset available for free access on the INEI website. INEI follows rigorous ethical standards as a public entity to protect the confidentiality of participants.

In this study, we adhere to the ethical principles of research, including the principles of intellectual honesty, truthfulness, transparency, human integrity, respect for intellectual property, fairness and accountability, in accordance with the “Code of Ethics in Research of the Universidad César Vallejo, version 01; by University Council Resolution N° 0340-2021-UCV”, using the data only for research purposes, as permitted by INEI’s terms of use. We have not attempted to re-identify any individual and our analysis does not include personally identifiable information.

## 3. Results

This article is based on the Tobit econometric model, an econometric model used to identify the impact of education on the gender wage gap in Peru during the period 2017-2021. This model used the database of the National Household Survey (ENAHO) 2017-2021, using the total wage (w_total) of men and women and explanatory variables such as age, number of children under 6 years old (under 6), years of education (educ) and children between 6 and 18 years old (between-6-18).

The gender gap in Peru showed a decreasing trend between men and women during the period 2017-2020, with an average reduction of 10% until the year 2020 due to the COVID-19 health crisis. The highest average salary achieved by men was in 2019, reaching S/. 2289.97 soles, while women reached an average salary of S/. 1368.85 soles. In the post-pandemic scenario for the year 2021, the gender gap increased by 3%, with men earning an average salary of S/. 1999.63 soles and women earning an average salary of S/. 1281.16 soles.

While the gender gap has clearly reduced, during the health crisis, men’s salaries showed a greater decline in 2020, with a negative variation of 16.5% compared to 2019. Meanwhile, women experienced a negative variation of only 11.5% during the health crisis compared to 2019. With the post-pandemic economic recovery, women’s salaries showed a more accelerated growth, reaching a variation of 5.7%, while men’s salaries grew by only 4.6%.

In Table 3, the Tobit model estimation shows that education has positively influenced the salaries of both men and women, with a clear wage gap of 137.56 soles. An increase of one year of education in men generates an increase in their salaries by 293.21 soles, while for women, an increase of one year of education generates an increase in their salaries by 155.65 soles.

**Table 3.** Tobit estimation of the monthly income gap between women and men in Peru, 2017.

		TOBIT		
Variables		Women		Men
		Marginal effect (%)		Marginal effect (%)
age	4.34	0.06	0.31	0.002
	(4.12)		(4.04)	
minor_6	16.00 **	0.22	-133.17 **	-0.96 **
	(84.48)		(64.52)	
between_6_18	4.49	0.06	8.12	0.06
	(43.48)		(37.37)	
**educ**	**155.65** ***	**2.14** ***	**293.21** ***	**2.11** ***
	(6.42)		(7.43)	
Constant	-372.86		-635.22 **	
	(249.07)		(250.77)	
Observations	4,429		15,313	
R-squared				

Note: Own elaboration with data from ENAHO INEI 2021, using Stata portable 16, 2022. Standard errors in parentheses.

*** p<0.01.

** p<0.05.

* p<0.1.

The marginal effect of the model shows that the probability of women’s incomes improving with an increase in years of education is 2.14%, while for men, the probability of their incomes improving with an increase in years of education is 2.11%, reflecting the greater impact of education on women. In the case of men, the probability of their incomes improving decreases by 0.96% with an increase in the number of children under 6 years old.

The Tobit model shows that the highly significant variables are “educ” (years of education) at 99% and “menor_6” (number of children under 6 years old) at 95%, demonstrating the positive impact of education on the gender wage gap in Peru in 2017.

In Table 4, the Tobit model estimation shows that education has positively influenced the salaries of both men and women, with a clear wage gap of 126.51 soles. An increase of one year of education in men generates an increase in their salaries by 286.30 soles, while for women, an increase of one year of education generates an increase in their salaries by 159.79 soles.

**Table 4.** Tobit estimation of the monthly income gap between women and men in Peru, 2018.

		TOBIT		
Variables		Women		Men
		Marginal effect (%)		Marginal effect (%)
age	9.87 ***	0.14 ***	-3.37	-0.03
	(3.69)		(3.78)	
minor_6	-96.49	-1.40	-153.62 **	-1.16 **
	(77.52)		(61.49)	
between_6_18	30.85	0.45	57.17	0.43
	(39.68)		(35.27)	
**educ**	**159.79** ***	**2.31** ***	**286.30** ***	**2.16** ***
	**(5.75)**		**(6.91)**	
Constant	-691.09 ***		-557.49 **	
	(224.66)		(235.35)	
Observations	4,791		15,953	
R-squared				

Note: Own elaboration with data from ENAHO INEI 2021, using Stata portable 16, 2022. Standard errors in parentheses.

*** p<0.01.

** p<0.05.

* p<0.1.

The marginal effect of the model shows that the probability of women’s incomes improving with an increase in years of education is 2.31%, while for men, the probability of their incomes improving with an increase in years of education is 2.16%, reflecting the greater impact of education on women. In the case of men, the probability of their incomes improving decreases by 1.16% with an increase in the number of children under 6 years old, and for women, the probability of their incomes improving with greater age is 0.14%.

Observing the Tobit model, the variables that are highly significant are “educ” (years of education) at 99%, “menor_6” (number of children under 6 years old) at 95%, and “edad” (age in years) at 99%, demonstrating the positive impact of education on the gender wage gap in Peru in 2018.

In Table 5, the Tobit model estimation shows that education has positively influenced the salaries of both men and women, with a clear wage gap of 128.83 soles. An increase of one year of education in men generates an increase in their salaries by 292.53 soles, while for women, an increase of one year of education generates an increase in their salaries by 163.70 soles.

**Table 5.** Tobit estimation of the monthly income gap between women and men in Peru, 2019.

		TOBIT		
Variables		Women		Men
	Coefficient of the variable (γkˆ)	Marginal effect (%)	Coefficient of the variable (γkˆ)	Marginal effect (%)
age	3.31	0.04	-5.01	-0.03
	(4.27)		(4.40)	
minor_6	-102.36	-1.33	-157.38 **	-1.08 **
	(90.05)		(73.91)	
between_6_18	-34.37	-0.45	4.26	0.03
	(46.67)		(42.03)	
**educ**	**163.70** ***	**2.12** ***	**292.53** ***	**2.01** ***
	(6.74)		(8.08)	
Constant	-308.63		-373.92	
	(263.17)		(275.98)	
Observations	4,613		14,350	
R-squared				

Note: Own elaboration with data from ENAHO INEI 2021, using Stata portable 16, 2022. Standard errors in parentheses.

*** p<0.01.

** p<0.05.

* p<0.1.

The marginal effect of the model shows that the probability of women’s incomes improving with an increase in years of education is 2.12%, while for men, the probability of their incomes improving with an increase in years of education is 2.01%, reflecting the greater impact of education on women. In the case of men, the probability of their incomes improving decreases by 1.08% with an increase in the number of children under 6 years old.

Observing the Tobit model, the variables that are highly significant are “educ” (years of education) at 99% and “menor_6” (number of children under 6 years old) at 95%, demonstrating the positive impact of education on the gender wage gap in Peru in 2019.

In Table 6, the Tobit model estimation shows that education has positively influenced the salaries of both men and women, with a clear wage gap of 106.18 soles. An increase of one year of education in men generates an increase in their salaries by 261.90 soles, while for women, an increase of one year of education generates an increase in their salaries by 155.72 soles.

**Table 6.** Tobit estimation of the monthly income gap between women and men in Peru, 2020.

		TOBIT		
Variables		Women		Men
	Coefficient of the variable (γkˆ)	Marginal effect (%)	Coefficient of the variable (γkˆ)	Marginal effect (%)
age	20.26 ***	0.31 ***	8.96 **	0.07 **
	(3.81)		(4.12)	
minor_6	-78.75	-1.19	-93.17	-0.74
	(76.54)		(67.48)	
between_6_18	76.68 *	1.16	-50.03	-0.40
	(41.59)		(38.35)	
**educ**	**155.72** ***	**2.35** ***	**261.90** ***	**2.09** ***
	(5.89)		(7.21)	
Constant	-1,325.34 ***		-1,062.85 ***	
	(230.85)		(252.35)	
Observations	4,352		13,127	
R-squared				

Note: Own elaboration with data from ENAHO INEI 2021, using Stata portable 16, 2022. Standard errors in parentheses.

*** p<0.01.

** p<0.05.

* p<0.1.

The marginal effect of the model shows that the probability of women’s incomes improving with an increase in years of education is 2.35%, while for men, the probability of their incomes improving with an increase in years of education is 2.09%, reflecting the greater impact of education on women. In the case of men, the probability of their incomes improving with an increase in age is 0.07%, and for women, the probability of their incomes improving with greater age is 0.31%.

Observing the Tobit model, the variables that are highly significant are “educ” (years of education) at 99% and “edad” (age in years) at 99%, demonstrating the positive impact of education on the gender wage gap in Peru in 2020.

In the Table 7, the Tobit model estimation shows that education has positively influenced the salaries of both men and women, with a clear wage gap of 101.17 soles. An increase of one year of education in men generates an increase in their salaries by 256.93 soles, while for women, an increase of one year of education generates an increase in their salaries by 155.76 soles.

**Table 7.** Tobit estimation of the monthly income gap between women and men in Peru, 2021.

		TOBIT		
Variables		Women		Men
		Marginal effect (%)		Marginal effect (%)
age	15.12 ***	2.12 ***	9.32 **	0.08
	(3.90)		(3.86)	
minor_6	-140.94 *	-1.92 **	-140.93 **	-1.16 **
	(78.16)		(64.84)	
between_6_18	61.40	0.84	76.10 **	0.63 **
	(42.23)		(37.00)	
**educ**	**155.76** ***	**2.12** ***	**256.93** ***	**2.12** ***
	(6.07)		(6.96)	
Constant	-990.24 ***		-1,060.74 ***	
	(236.14)		(237.57)	
Observations	5,251		12,678	
R-squared				

Note: Own elaboration with data from ENAHO INEI 2021, using Stata portable 16, 2022. Standard errors in parentheses.

*** p<0.01.

** p<0.05.

* p<0.1.

The marginal effect of the model shows that the probability of women’s incomes improving with an increase in years of education is 2.12%, which is the same as the probability for men. For women, the probability of their incomes improving with an increase in age is 2.12%, while for men, the probability of their incomes improving decreases by 1.16% with an increase in the number of children under 6 years old, and for women, the probability decreases by 1.92%.

Observing the Tobit model, the variables that are highly significant are “educ” (years of education) at 99%, “menor_6” (number of children under 6 years old) at 95%, “entre_6_18” (number of children aged between 6 and 18) at 95%, and “edad” (age in years) at 95%, demonstrating the positive impact of education on the gender wage gap in Peru in 2021.

## 4. Discussion

The previously found results show that during the analysis period of 2017-2021, years of education have had a positive impact on the gender wage gap in Peru based on the Tobit estimation. These findings are consistent with those of Picatoste et al. (2023) which revealed that the gender gap is a relevant aspect of the European Union, considering that education has significantly influenced the wage gap and is a relevant value in the social aspect.

Similarly, they are related to Reshi and Sudha (2023) whose findings show that the gender wage gap has a significant impact on women’s empowerment due to the limitations of women’s economic independence and autonomy.

On the other hand, Penner et al. (2022) in their various studies, indicate that the gender wage gap highlights that various countries can be identified where the wage gap has been a crucial aspect of gender differences due to different jobs that substantially represent wage differences between men and women, developing wage inequality that faces various challenges in comparing results.

Adriaans and Targa (2023) reveal that women with higher education levels tend to earn higher wages compared to those with lower education levels. However, the gender wage gap persists even among people with similar education levels.

Differences in labor market participation between men and women, such as the proportion of women working full-time versus part-time, can contribute to the wage gap.

The increase in women’s participation in higher education and in academic and professional fields traditionally dominated by men can have a positive impact on reducing the wage gap.

In this way, Hutter and Weber (2023) consider that growing economic inequality has highlighted a variety of literature where wage dispersion involves significant inequality using the VECM model. Considering the structural model reveals that it affects inequality with various impacts on the labor market and becomes relevant in wages with a biased technological shock and structural inequality that negatively impacts working hours and reduces productivity by facing negative inequality in the labor market characterized by high levels of inequality.

Accessible and equitable education provides men and women with the same opportunities to acquire skills and knowledge. This establishes the foundation for fair labor competition and reduces initial disparities that could contribute to wage gaps.

Education provides people with the necessary skills and competencies to perform different labor roles. Acquiring specific skills and advanced knowledge can increase an employee’s perceived value, regardless of gender.

Solid education allows people to make informed career decisions. Encouraging women to enter fields traditionally dominated by men and men to consider options in predominantly female fields can contribute to reducing wage gaps.

Additionally, quality education can offer opportunities for social and economic mobility, allowing people to overcome socioeconomic barriers. This is especially important for groups that have historically faced discrimination, such as women, as education can be a vehicle for empowerment and financial autonomy.

## 5. Conclusions

Gender wage gap in Peru has shown a decreasing trend between men and women from 2017 to 2020 during the health crisis scenario, only to be reversed in the post-pandemic period of 2021, where the gender gap grew by 3%, with men reaching an average salary of S/.1999.63 soles and women reaching an average salary of S/.1281.16 soles. Despite this, women exhibited a higher accelerated salary growth, with a variation of 5.7%, while men’s salaries only grew by 4.6% in the economic reactivation of 2021 compared to 2020.

The Tobit model estimation in 2017 considers the variables of education (years of education) significant at 99%, minor_6 (number of children under 6 years old) at 95%, and age (years) at 99%. In 2018, the highly significant variables are education (years of education) at 99% and minor_6 (number of children under 6 years old) at 95%. In contrast, in 2019, the highly significant variables are education (years of education) at 99% and age (years) at 99%, and for 2020, the highly significant variables are education (years of education) at 99%, minor_6 (number of children under 6 years old) at 95%, between_6_18 (number of children aged between 6 and 18 years) at 95%, and age (years) at 95%; with education years being highly significant for both men and women at 99% in influencing their salaries during the period 2017-2021.

The Tobit model estimation shows that in 2019, women generated an increase in their salaries by S/.163.70 soles for every additional year of education, while in 2017, men generated an increase in their salaries by S/.293.21 soles for every additional year of education.

During the analysis period of 2017-2021, years of education have had a positive impact on the gender wage gap in Peru, with the greatest impact during the health crisis, as the probability of women’s incomes improving with an increase in years of education was 2.35%, and for men, their greatest impact was in 2018, with a probability of their incomes improving with an increase in years of education at 2.16% in terms of marginal effect.

In conclusion, it is emphasized that the findings recommend promoting gender equality in both access to education and professional training for women. Additionally, states should implement appropriate policies to support salary equities and job opportunities for both men and women.

## Data Availability

*Zenodo.* Evaluation of the Impact of the Educational Revolution in Peru and the Gender Wage Gap, 2017-2021, https://doi.org/10.5281/zenodo.12772670 ( Castro et al., 2024). This project contains the following underlying data:
• Data_Dynamics of the Gender Wage Gap in the Period 2017-2021. •
Figure 1_Dynamics of the Gender Wage Gap in the Period 2017-2021. Data is available under Creative Commons Zero v1.0 Universal *Zenodo.* Evaluation of the Impact of the Educational Revolution in Peru and the Gender Wage Gap, 2017-2021. https://doi.org/10.5281/zenodo.12772670 ( Castro et al., 2024). This project contains the following extended data:
• ModelTOBIT 2021. • License_Stata 16 software. •
Strobe_checklist_v4_combined. • Rectoral Resolution N° 760-2007_UCV_CODE OF ETHICS. Data is available under Creative Commons Zero v1.0 Universal

## References

[ref1] Aarsman SR Greenwood CJ Linardon J : Enhancing inferences and conclusions in body image focused non-experimental research via a causal modelling approach: A tutorial. *Body Image.* 2024;49(February):101704. 38579514 10.1016/j.bodyim.2024.101704

[ref2] Abdel-Rahman S Awwad FA Qasim M : New evidence of gender inequality during COVID-19 outbreak in the Middle East and North Africa. *Heliyon.* 2023;9(7):e17705. 37456038 10.1016/j.heliyon.2023.e17705 PMC10338972

[ref3] Adriaans J Targa M : Gender differences in fairness evaluations of own earnings in 28 European countries. *Eur. Soc.* 2023;25(1):107–131. 10.1080/14616696.2022.2083651

[ref4] Afrin S Shammi M : A review on the gendered impact of COVID-19 pandemic towards achieving sustainable development goals in Bangladesh: Ecofeminist perspectives on the response to COVID-19 pandemic. *Heliyon.* 2023;9(3):e14680. 36967926 10.1016/j.heliyon.2023.e14680 PMC10027295

[ref5] Alwago WO : A partial least squares analysis of gender inequality, occupational segregation, and economic growth: Evidence from Sub-Saharan Africa. *Reg. Sci. Policy Pract.* 2023;15(6):1299–1317. 10.1111/rsp3.12677

[ref6] Barra Quispe DE : Factores que influyen sobre la brecha salarial por género de los trabajadores dependientes en el Perú, 2012-2016. 2018. Reference Source

[ref7] Bataka H : Global value chains participation and gender inequalities in Sub-Saharan Africa: Importance of women education. *Int. Econ.* 2024;178(February):100483. 10.1016/j.inteco.2024.100483

[ref8] Brzezinski M : The impact of past pandemics on economic and gender inequalities. *Econ. Hum. Biol.* 2021;43(July):101039. 34247056 10.1016/j.ehb.2021.101039

[ref9] Castro P Morán R Llonto Y : Evaluation of the impact of the educational revolution in Peru and the gender wage gap, 2017-2021.[Data set]. *Zenodo.* 2024. 10.5281/zenodo.12772670

[ref10] Carlosviza M Collantes I Agurto B : La Lucha contra la Brecha de Género Salarial en tiempos de pandemia. Un análisis para el caso peruano. *Económica.* 2021;8:149–165. Reference Source

[ref11] Castagnetti C Giorgetti ML : Understanding the gender wage-gap differential between the public and private sectors in Italy: A quantile approach. *Econ. Model.* 2019;78(September 2018):240–261. 10.1016/j.econmod.2018.09.025

[ref12] Chowdhury I Johnson HC Mannava A : Gender Gap in Earnings in Vietnam Why Do Vietnamese Women Work in Lower Paid Occupations? *Policy Research Working Paper.* 2018; pp.1–40. Reference Source

[ref13] Costas A : A new post-pandemic social contract. The role of the Social Economy. *CIRIEC-Espana Revista de Economia Publica, Social y Cooperativa.* 2020;100:11–29. 10.7203/CIRIEC-E.100.18715

[ref14] Da Costa Silva MM Shinkoda MH : The gender gap and the COVID-19 pandemic: An analysis of net Brazilian formal job destruction. *Economia.* 2021;22(3):225–238. 10.1016/j.econ.2021.12.002

[ref15] Dancausa MG Millán MG Hernández R : The spanish labor market: A gender approach. *Int. J. Environ. Res. Public Health.* 2021;18(5):1–23. 33800434 10.3390/ijerph18052742 PMC7967455

[ref16] Defensoría del Pueblo: El impacto económico de la brecha salarial por razones de género. 2019. Reference Source

[ref17] Designed Research; A, S. S. D. S Performed Research; A, S. S. D. S: The confidence gap predicts the gender pay gap among STEM graduates. *Proc. Natl. Acad. Sci. U S A.* 2010;117(48):30303–30308. 33199594 10.1073/pnas.2010269117/-/DCSupplemental PMC7720106

[ref18] Díaz FS : Evolution and analysis of salary discrimination by gender in Chile. *Trimestre Economico.* 2021;88(349):39–75. 10.20430/ETE.V88I349.984

[ref19] Didier N : Does the expansion of higher education reduce gender gaps in the labor market? Evidence from a natural experiment. *Int. J. Educ. Dev.* 2021;86(July):102467. 10.1016/j.ijedudev.2021.102467

[ref20] England P Levine A Mishel E : Progress toward gender equality in the United States has slowed or stalled. *Proc. Natl. Acad. Sci. USA.* 2020;117(13):6990–6997. 32229559 10.1073/pnas.1918891117 PMC7132302

[ref21] Fox D Gmeiner M Price J : The gender gap in K-12 educator salaries. *Econ. Educ. Rev.* 2019;68:23–26. 10.1016/j.econedurev.2018.11.004

[ref22] Gonzáles NN : Impacto de política de igualdad de género para el acceso de las mujeres al mercadp de trabajo en el Perú 2007-2014. 2021. Reference Source

[ref23] Hutter C Weber E : Labour market effects of wage inequality and skill-biased technical change. *Appl. Econ.* 2023;55(27):3063–3084. 10.1080/00036846.2022.2108751

[ref24] INEI: National Household Survey from 2017 to 2021. 2024. Reference Source

[ref25] Keller W Molina T Olney WW : The gender gap among top business executives. *J. Econ. Behav. Organ.* 2023;211:270–286. 10.1016/J.JEBO.2023.04.017

[ref26] Kireyeva A Satybaldin A : Analysis of Gander Pay Gap in Different Sectors of the Economy in Kazakhstan. *Asian Finance, Economics and Business.* 2019;6(2):231–238. 10.13106/jafeb.2019.vol6.no2.231

[ref27] Kurek B Górowski I : Gender and Age as Determinants of Expected Rate of Return on Human Capital. *Cent. Eur. Manag. J.* 2020;28(4):30–50. 10.7206/cemj.2658-0845.33

[ref28] Leibing A Peter F Waights S : Gender gaps in early wage expectations. *Econ. Educ. Rev.* 2023;94(June 2022):102398. 10.1016/j.econedurev.2023.102398

[ref29] Litman L Robinson J Rosen Z : The persistence of pay inequality: The gender pay gap in an anonymous online labor market. *PLoS One.* 2020;15(2):e0229383. 32084233 10.1371/journal.pone.0229383 PMC7034870

[ref30] Mandel H Rotman A : Revealing the concealed effect of top earnings on the gender gap in the economic value of higher education in the united states, 1980–2017. *Demography.* 2021;58(2):551–570. 33834232 10.1215/00703370-9009367

[ref31] Maxwell JA Reybold LE : Qualitative Research. *International Encyclopedia of the Social & Behavioral Sciences: Second Edition.* Elsevier;2015; Vol.19. 10.1016/B978-0-08-097086-8.10558-6

[ref32] Nguyen CP : Uncertainty and gender inequality: A global investigation. *Q. Rev. Econ. Finance.* 2022;86:31–47. 10.1016/j.qref.2022.06.003

[ref33] Ñiquen O : El impacto del nivel educativo alcanzado en el índice de calidad del empleo en el Perú, 2016. *Revista Peruana de Investigación Educativa.* 2019;11:5–38. 10.34236/rpie.v11i11.91 Reference Source

[ref34] Organización Internacional de Trabajo: La brecha salarial entre hombres y mujeres en América Latina. 2019. Reference Source

[ref35] Penner AM Petersen T Hermansen AS : Within-job gender pay inequality in 15 countries. *Nat. Hum. Behav.* 2022;7(2):184–189. 36424396 10.1038/s41562-022-01470-z PMC9957724

[ref36] Picatoste X Mesquita A González-Laxe F : Gender wage gap, quality of earnings and gender digital divide in the European context. *Empirica.* 2023;50(2):301–321. 10.1007/S10663-022-09555-8/TABLES/5 PMC9714396 36471732

[ref37] Quadlin N VanHeuvelen T Ahearn CE : Higher education and high-wage gender inequality. *Soc. Sci. Res.* 2023;112(February):102873. 37061326 10.1016/j.ssresearch.2023.102873 PMC10712336

[ref38] Reissová A Šimsová J : The value of education in the labour market. How realistic are student expectations? 2019. 10.15208/beh.2019.2

[ref39] Reshi IA Sudha DT : The Gender Pay Gap And Its Impact On Women’s Economic Empowerment. *Morfai Journal.* 2023;3(1):9–16. 10.54443/Morfai.V3i1.742

[ref40] Rios PO : Educación y Brecha Salarial de Género en el Perú. 2019. Reference Source

[ref41] Schober T Winter-Ebmer R : Gender wage inequality and economic growth: Is there really a puzzle?-A comment. *World Dev.* 2011;39(8):1476–1484. 21857765 10.1016/j.worlddev.2011.05.001 PMC3145160

[ref42] Singh V Shirazi H Turetken J : COVID-19 and gender disparities: Labour market outcomes. *Res. Econ.* 2022;76(3):206–217. 35909917 10.1016/j.rie.2022.07.011 PMC9327182

[ref43] Smith T Cecala A Penner M : It’s Not Just a Pay Gap: Quantifying the Gender Wage and Pension Gap at a Post-Secondary Institution in Canada. *Can. J. High. Educ.* 2021;51(51):74–84. 10.47678/cjhe.vi0.189215 Reference Source

[ref44] Sterling AD Thompson ME Wang S : The confidence gap predicts the gender pay gap among STEM graduates. *Proc. Natl. Acad. Sci. USA.* 2020;117(48):30303–30308. 33199594 10.1073/pnas.201026911 PMC7720106

[ref45] Sugiharti RR Kurnia AS : Gender Wage Gap and Education: Case in Indonesia’s Labor Market. *E3S Web Conf.* 2018;73:1–4. 10.1051/e3sconf/20187311019

[ref46] Suharyono Digdowiseiso K : Education and gender wage gap: Evidence from indonesia. *Account. Forum.* 2021;7(1):33–40. 10.5267/j.ac.2020.10.017

[ref47] Tansel A Keskin HI Ozdemir ZA : Public-private sector wage gap by gender in Egypt: Evidence from quantile regression on panel data, 1998–2018. *World Dev.* 2020;135:105060. 10.1016/j.worlddev.2020.105060

[ref48] Valdez RI Sobrevilla V : The sectoral-regional structure of the wages in mexico. *Papeles de Poblacion.* 2021;27(108):185–209. 10.22185/24487147.2021.108.16

[ref49] Zhou X Pan G : Higher Education and the Black-White Earnings Gap. *Am. Sociol. Rev.* 2023;88(1):154–188. 10.1177/00031224221141887

