# Supplementary material for: Evaluation of the impact of the educational revolution in Peru and the gender wage gap, 2017-2021
Source: F1000Res. 2024 Aug 5;13:884. [Version 1] doi: 10.12688/f1000research.153475.1 (PMC13680836; doi:10.12688/f1000research.153475.1)
Supplement: Peer review history

# Reviewer report: Reviewer response for version 1

**DOI:** 10.5256/f1000research.168385.r489354

**Published:** 2026-10-19

**Authors:** Rosmery Sabina Pozo Enciso

**Affiliations:** 1 Universidad Nacional Micaela Bastidas de Apurimac, Abancay, Apurimac, Peru

Following a comprehensive review of the manuscript “Evaluation of the impact of the educational revolution in Peru and the gender wage gap, 2017-2021” the article is considered to meet the academic, methodological, and technical standards required for acceptance. The study addresses a relevant economic and social issue: the influence of education on the gender wage gap in Peru during the 2017–2021 period.

 INTRODUCTION

 The Introduction provides an appropriate account of the research problem and substantiates the relationship among education, human capital, economic inequality, and the gender wage gap. It also incorporates national and international studies that establish the relevance of the topic and justify the research objective. Although some arguments could be organized more concisely and the study’s specific contribution could be highlighted more precisely, the literature review is sufficient to support the study.

 METHODOLOGY

 The methodology is consistent with the stated objective. The article adopts a quantitative approach, an explanatory scope, and a nonexperimental design, using a Tobit econometric model to estimate the influence of education on labor income. The data were obtained from the housing, household-member characteristics, education, employment, and income modules of Peru’s National Household Survey for the 2017–2021 period.

 The study population and the inclusion and exclusion criteria are described with sufficient detail. In addition, the numbers of observations for men and women are reported for each year analyzed, which facilitates an understanding of the size and composition of the sample.

 RESULTS

 The Results section is logically organized, beginning with a descriptive analysis of income trends and continuing with econometric estimates disaggregated by sex and year. The findings show that years of education have a positive and statistically significant influence on the income of both men and women, although differences remain in the magnitude of the estimated coefficients.

 The tables report coefficients, marginal effects, standard errors, significance levels, and numbers of observations. For example, in 2020, the marginal effect of education was 2.35% for women and 2.09% for men, whereas in 2021 the estimated marginal effect was 2.12% for both groups.

 DISCUSSION

 The Discussion relates the findings to previous research on education, labor-force participation, wage inequality, and women’s economic autonomy. The conclusions address the stated objective and are supported by the reported estimates, particularly regarding the importance of education and the persistence of wage differences between men and women.

 The article also provides information to facilitate reproducibility. The data were drawn from ENAHO, a publicly available source, and the authors declare that the underlying data, the Tobit model, the STROBE checklist, and other supplementary documents are available through a Zenodo repository.

 FINAL RECOMMENDATION

 ACCEPTED

 Overall, the article has a clear structure, an appropriate methodology, consistent results, and conclusions aligned with the evidence obtained. The study makes a relevant contribution to the analysis of education, labor income, and the gender wage gap in the Peruvian context. Accordingly, acceptance for indexing is recommended

Is the work clearly and accurately presented and does it cite the current literature?

Yes

If applicable, is the statistical analysis and its interpretation appropriate?

Yes

Are all the source data underlying the results available to ensure full reproducibility?

Yes

Is the study design appropriate and is the work technically sound?

Yes

Are the conclusions drawn adequately supported by the results?

Yes

Are sufficient details of methods and analysis provided to allow replication by others?

Yes

Reviewer Expertise:

PhD in Education, with Master’s degrees in Foreign Language Teaching and Social Management, a Bachelor’s degree in Education and a law degree.

I confirm that I have read this submission and believe that I have an appropriate level of expertise to confirm that it is of an acceptable scientific standard.

# Reviewer report: Reviewer response for version 1

**DOI:** 10.5256/f1000research.168385.r320571

**Published:** 2025-09-16

**Authors:** Ricardo Fernando Cosio Borda

**Affiliations:** 1 Universidad EAN, Bogotá, Bogota, Colombia

The introduction is acceptable, although it could have been strengthened with a more thorough review of the literature. However, it is sufficient to theoretically support the nature of the research.

 The paper shows methodological strengths, clearly describing the process for data collection and analysis. The study sample has been clearly described and specified.

 The results show an initial descriptive analysis followed by the application of the econometric model. This section is acceptable, although the information in the descriptive statistical analysis could have been exploited further.

 The discussion and conclusions are relevant.

 Overall, the paper makes an acceptable contribution and meets the necessary criteria for acceptance.

Is the work clearly and accurately presented and does it cite the current literature?

Yes

If applicable, is the statistical analysis and its interpretation appropriate?

Yes

Are all the source data underlying the results available to ensure full reproducibility?

Yes

Is the study design appropriate and is the work technically sound?

Yes

Are the conclusions drawn adequately supported by the results?

Yes

Are sufficient details of methods and analysis provided to allow replication by others?

Yes

Reviewer Expertise:

Social sciences and economic studies.

I confirm that I have read this submission and believe that I have an appropriate level of expertise to confirm that it is of an acceptable scientific standard.
